# Fresh and frozen cardiac tissue are comparable in DNA methylation array *β*-values, but formalin-fixed, paraffin-embedded tissue may overestimate DNA methylation levels

**DOI:** 10.1038/s41598-023-43788-2

**Published:** 2023-09-29

**Authors:** Mikkel Eriksen Dupont, Stine Bøttcher Jacobsen, Steffan Noe Niikanoff Christiansen, Jacob Tfelt-Hansen, Morten Holdgaard Smerup, Jeppe Dyrberg Andersen, Niels Morling

**Affiliations:** 1 Section of Forensic Genetics, Department of Forensic Medicine, Faculty of Health and Medical Sciences, University of Copenhagen, Copenhagen, Denmark; 2 Department of Health Science and Technology, Aalborg University, Aalborg, Denmark; 3 Department of Cardiology, Rigshospitalet, Copenhagen University Hospital, Copenhagen, Denmark; 4 Department of Cardiothoracic Surgery, Rigshospitalet Copenhagen University Hospital, Copenhagen, Denmark

**Keywords:** Epigenetics, DNA methylation, Epigenetics, Cardiovascular diseases, Cardiovascular genetics, Cardiology, Biological techniques, Bioinformatics, Epigenetics analysis, Genetic techniques

## Abstract

Untreated fresh cardiac tissue is the optimal tissue material for investigating DNA methylation patterns of cardiac biology and diseases. However, fresh tissue is difficult to obtain. Therefore, tissue stored as frozen or formalin-fixed, paraffin-embedded (FFPE) is widely used for DNA methylation studies. It is unknown whether storage conditions alter the DNA methylation in cardiac tissue. In this study, we compared the DNA methylation patterns of fresh, frozen, and FFPE cardiac tissue to investigate if the storage method affected the DNA methylation results. We used the Infinium MethylationEPIC assay to obtain genome-wide methylation levels in fresh, frozen, and FFPE tissues from nine individuals. We found that the DNA methylation levels of 21.4% of the examined CpG sites were overestimated in the FFPE samples compared to that of fresh and frozen tissue, whereas 5.7% were underestimated. Duplicate analyses of the DNA methylation patterns showed high reproducibility (precision) for frozen and FFPE tissues. In conclusion, we found that frozen and FFPE tissues gave reproducible DNA methylation results and that frozen and fresh tissues gave similar results.

## Introduction

Large-scale genetic studies are important for identifying cardiac disease risk genes and molecular pathways involved in disease development^1–3^. Cardiac diseases are associated with lifestyle factors that may affect the molecular mechanisms of the cells by altering gene expression through epigenetic regulation^4^. One of the most well-studied epigenetic regulations is DNA methylation, the covalent addition of a methyl group primarily to cytosines followed by a guanine nucleotide (CpG). DNA methylation is essential for cellular functions such as differentiation, genomic imprinting, and X chromosome inactivation^5^. DNA methylation patterns are cell-type specific, and to understand how methylation patterns affect cardiac diseases, studies of DNA methylation in cardiac tissues are essential.

DNA methylation can be investigated using various methods^6,7^. The Infinium MethylationEPIC assay (EPIC array) (Illumina, Inc., CA, USA) is an array-based probe hybridization method for studying DNA methylation levels at more than 850,000 sites throughout the human genome. The assay and its predecessor Illumina HumanMethylation450k have been widely used for DNA methylation analysis due to the high number of examined methylation sites for a reasonable price^8^. The EPIC array requires bisulfite treatment of the DNA to assess the methylation status of the cytosines. The bisulfite conversion is harsh to the DNA, which therefore must be of a reasonable quality for successful analysis^9^.

Fresh cardiac tissue is rarely readily available, and DNA methylation studies of cardiac tissues are mainly done on stored biopsies from surgeries and autopsies^10^. Storage, however, potentially affects the tissue and DNA. A common way to preserve tissue for storage at clinical and forensic pathology laboratories is formalin-fixation, paraffin-embedding (FFPE)^11^. Formalin fixation preserves tissue morphology and is ideal for subsequent histopathological and immunohistochemical investigations^12^. FFPE allows inexpensive storage at room temperature of extensive tissue archives that represent vast resources for retrospective disease studies. A downside to FFPE is that formalin cross-links nucleotides and proteins, resulting in DNA degradation and lower DNA quality^13–15^.

When tissues are stored by freezing at -80°C, many of the disadvantages of FFPE are avoided, and it is considered the standard storage method for DNA analysis, including DNA methylation. The impact of freezing and FFPE on the results of DNA methylation array data has been observed for tissues such as brain, breast, and ovarian tumours^16–18^. These studies showed correlations of DNA methylation levels among paired frozen and FFPE tissue samples ranging from *r*^*2*^ = 0.90 to *r*^*2*^ = 0.99. To our knowledge, similar studies have not yet been performed on cardiac tissue. Furthermore, no study has investigated how freezing and FFPE of samples influence the methylation patterns by comparing DNA methylation levels to those obtained from fresh untreated material. With this study, we aim to investigate if cardiac tissue stored under different conditions (freezing and FFPE) produce similar and reproducible DNA methylation results by comparing the result to those obtained from fresh cardiac tissues. This is important to investigate as fresh cardiac tissues is often not available and the preferred storage methods for archived tissues are freezing and FFPE.

**Figure 1:**
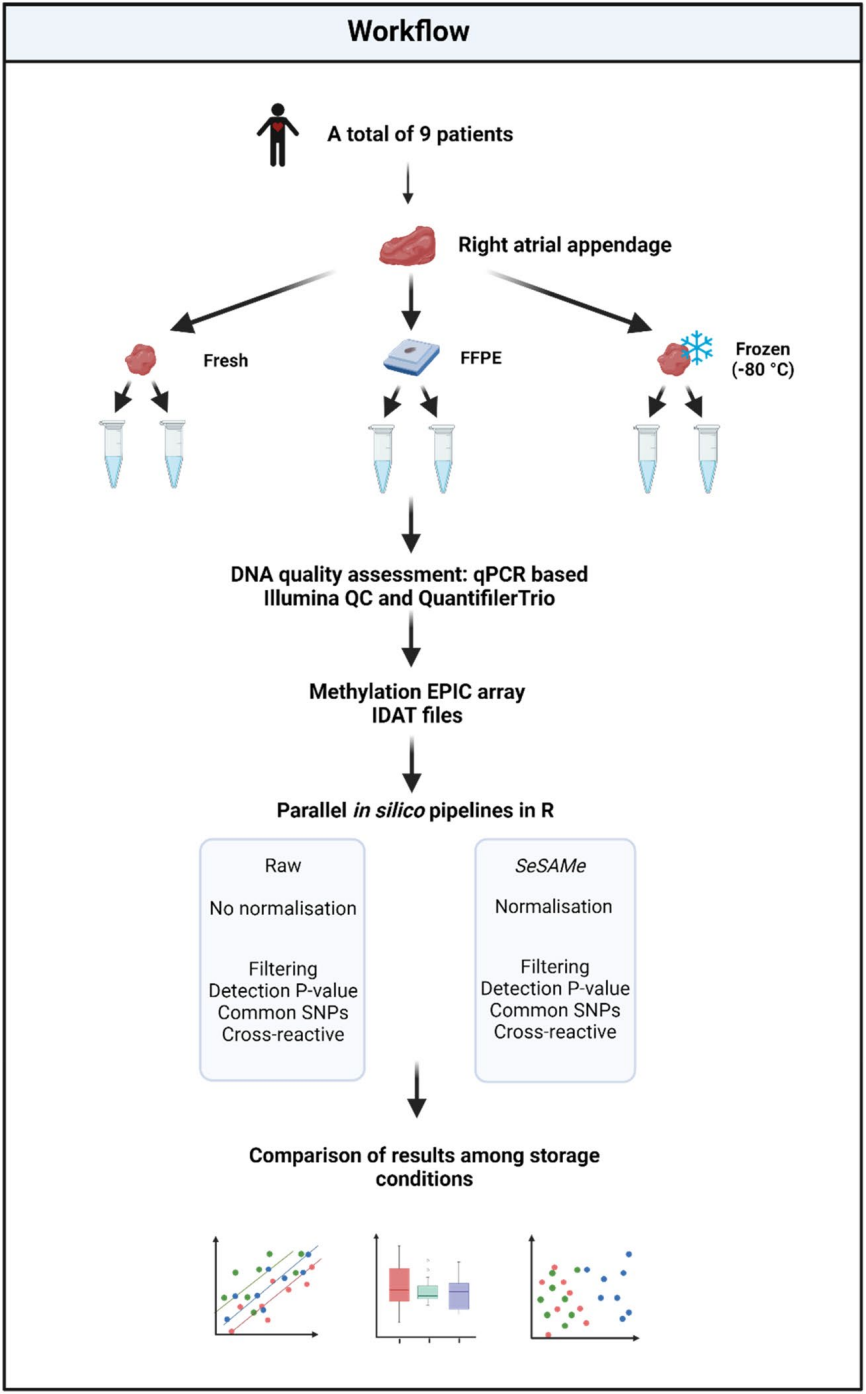
Graphical representation of the workflow. Raw data was imported by the R package *minfi*^20^ using *preprocessRaw()* function. *SeSAMe* data was imported using the standard preprocessing settings. QC = Quality control, FFPE = Formalin-fixed, paraffin-embedded tissue. Created with Biorender.com.

We performed duplicate EPIC array DNA methylation analysis of paired fresh, frozen, and FFPE cardiac tissues collected from surgeries to investigate the impact of storage conditions on DNA methylation levels (Fig. 1). We evaluated the quality of the extracted DNA and assessed how the storage conditions affected the EPIC array results. The EPIC array data were assessed both as raw data and as normalized data using the standard settings of a *SeSAMe* (SEnsible Step-wise Analysis of DNA MEthylation BeadChips) pipeline^19^. The reproducibility of the results with each storage condition was assessed by evaluating the correlation of methylation levels (*β*-values) between duplicate samples. Furthermore, *β*-values were compared among fresh, frozen, and FFPE tissues at the individual CpG level and the overall DNA methylation.

## Results

**Figure 2:**
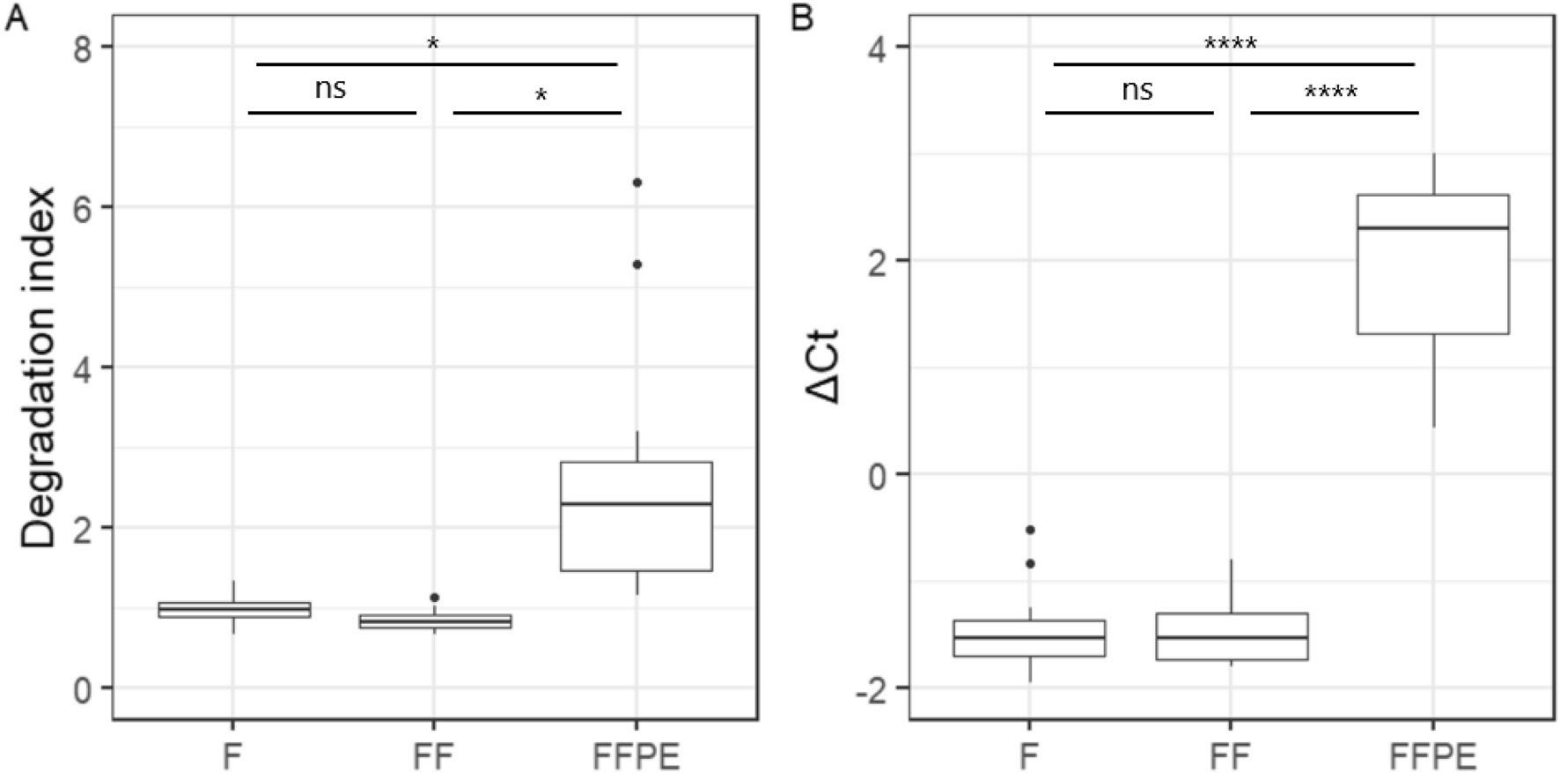
Quality of DNA extracted from fresh (F), frozen (FF), and formalin-fixed, paraffin-embedded (FFPE) cardiac tissue. (**A**) Box plot of degradation index measured with Quantifiler Trio (n = 54, duplicates of 9 patient samples in each group). (**B**) Box plot of ΔCt measured with Infinium QC (n = 54, duplicates of 9 patient samples in each group). Paired students t-tests, ns = non significant, * = p < 0.5, **** = p < 0.0001.

The quality of the DNA extracted from fresh, frozen, and FFPE tissue was evaluated by the qPCR-based methods The Quantifiler™ Trio DNA Quantification Kit (Quantifiler Trio) and the Infinium HD FFPE QC kit (Infinium QC). The larger the degradation index (DI) of Quantifiler Trio and ΔCt of the Infinium QC, the lower quality of DNA. The FFPE tissue was found to have statistically significantly higher DI (mean = 2.51) than fresh (mean = 0.97, p < 0.05) and frozen tissues (mean = 0.84, p < 0.05) (Fig. 2A). The ΔCt of FFPE tissue (mean = 2.03) was statistically significantly higher than those of fresh (mean = − 1.47, p < 1 × 10^–6^) and frozen (mean = − 1.46, p < 1 × 10^–6^) tissues (Fig. 2B).

**Figure 3:**
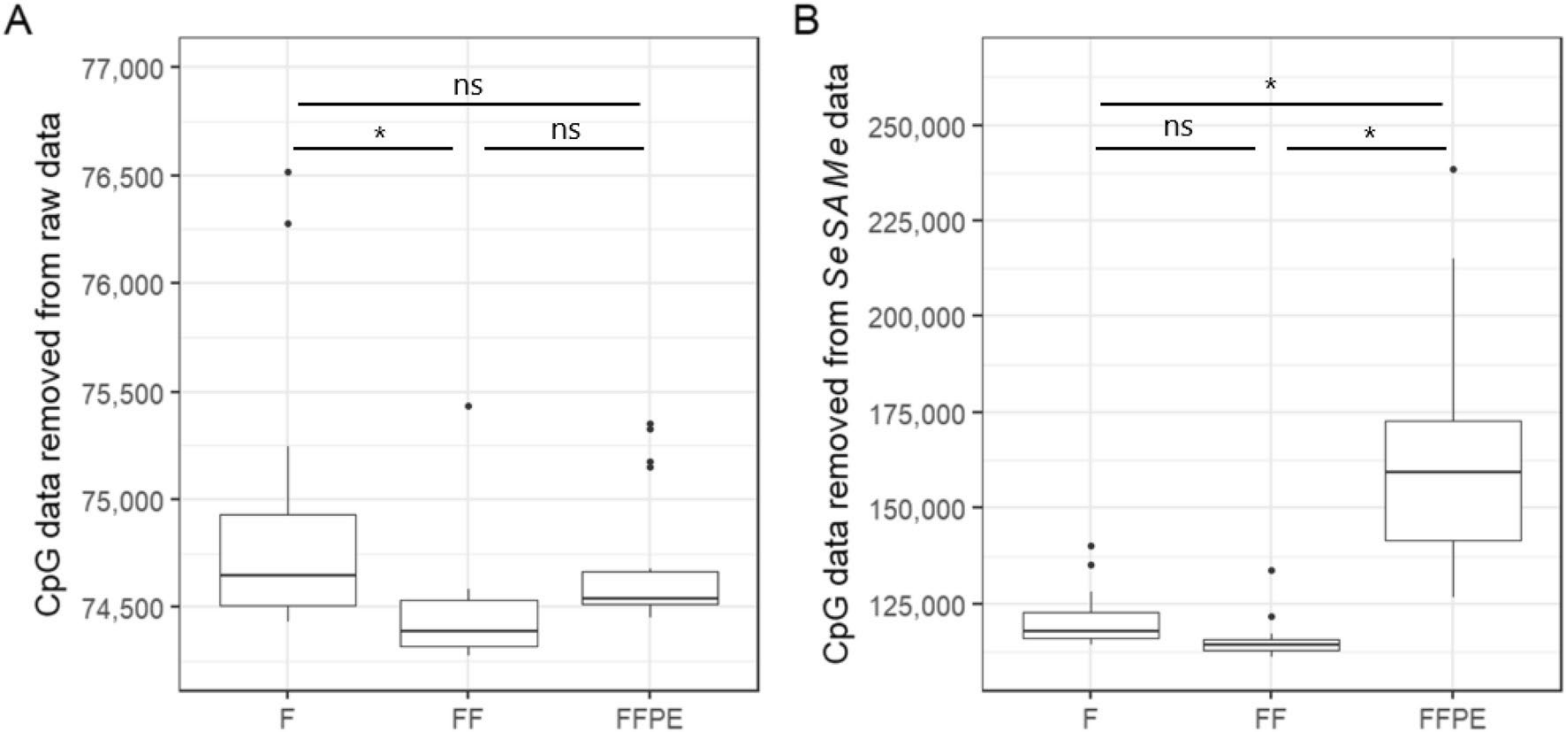
CpG probe data removed from sample data after in silico quality control (QC). Box plots of CpG probe data filtered and removed per sample from the raw (**A**) data (n = 54, duplicates of 9 patient samples in each group) and *SeSAMe* processed (**B**) data (n = 54, duplicates of 9 patient samples in each group). F = Fresh tissue, FF = Frozen tissue, FFPE = Formalin-fixed, paraffin-embedded tissue. Paired Wilcoxon’s rank-sum tests, ns = non significant, * = p < 0.05.

From the raw intensity data, the in silico quality control (QC) step filtered and removed a median of 74,646 (8.6%) CpG probes for fresh, 74,386 (8.6%) CpG probes for frozen, and 74,542 (8.6%) CpG probes for FFPE samples (Fig. 3A). Fresh tissue had statistically significantly more CpG probe data removed than frozen tissue (p < 0.05). Analysing the intensity data with the *SeSAMe* pipeline the in silico QC filtered and removed a median of 117,737 (13.6%) CpG probes for fresh, 114,420 (13.2%) CpG probes for frozen, and 159,178 (18.4%) for FFPE tissue (Fig. 3B). Statistically significantly more CpG probe data were removed from FFPE tissue than fresh and frozen tissues (p < 0.05).

**Figure 4:**
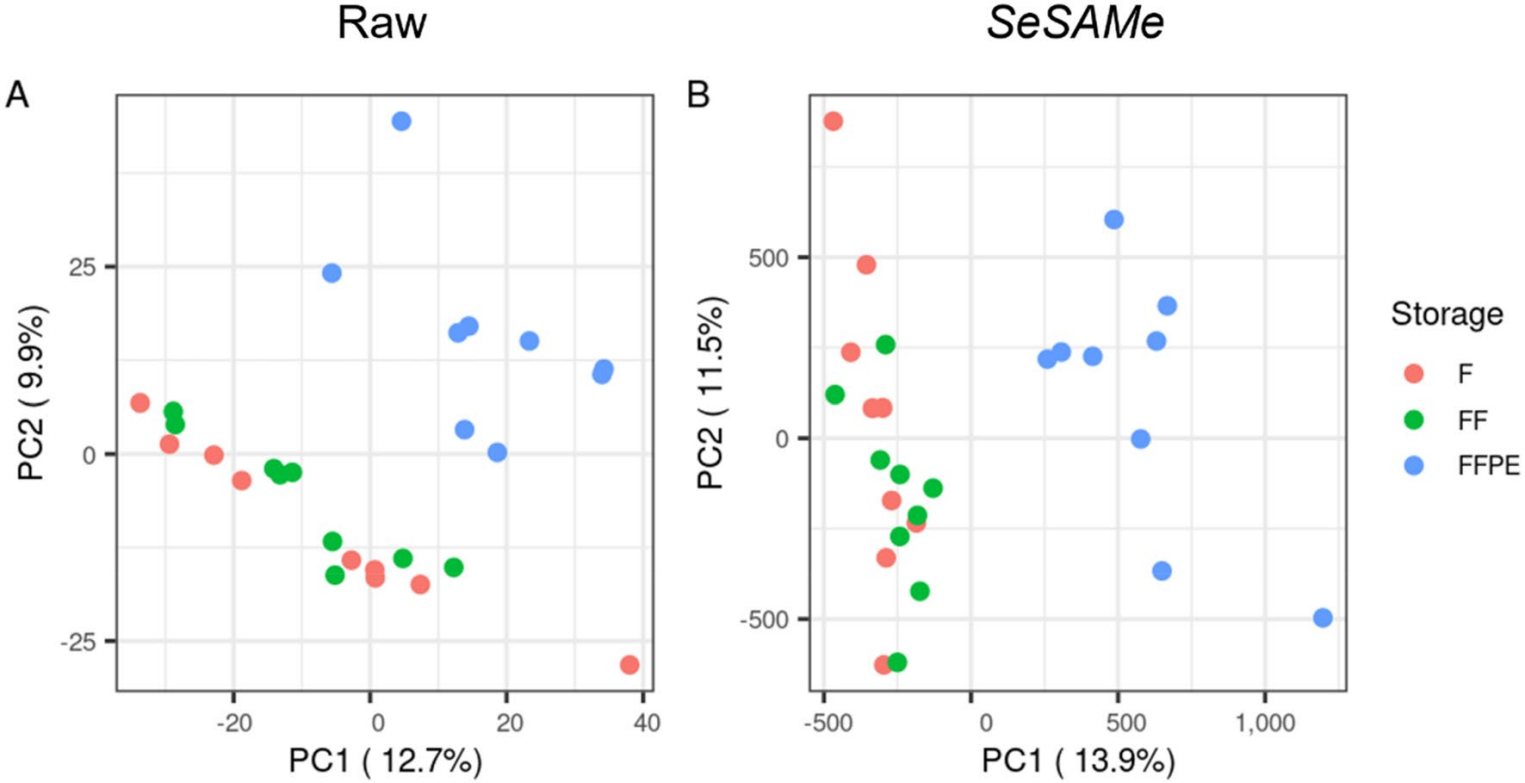
Principal component analyses of *ß*-values. (**A**) Raw duplicate mean *ß*-values (n = 27, 9 patient samples in each group). (**B**) *SeSAMe* duplicate mean *ß*-values (n = 27, 9 patient samples in each group). F = Fresh tissue, FF = Frozen tissue, FFPE = Formalin-fixed, paraffin-embedded tissue.

To identify similarities across the datasets, principal component analyses (PCA) were carried out with raw and *SeSAMe ß*-values (Fig. 4). Both PCAs showed that the FFPE samples clustered separately from those of fresh and frozen samples. The first and second principal components (PC1 and PC2) explained 12.7% and 9.9%, respectively, of the total variation of the raw *ß*-values (Fig. 4A) and 13.9% and 11.5%, respectively, of the *SeSAMe ß*-values (Fig. 4B). PC1 and PC2 showed no clear separation caused by intra-individual variation (Supplementary Figure S1).

**Figure 5:**
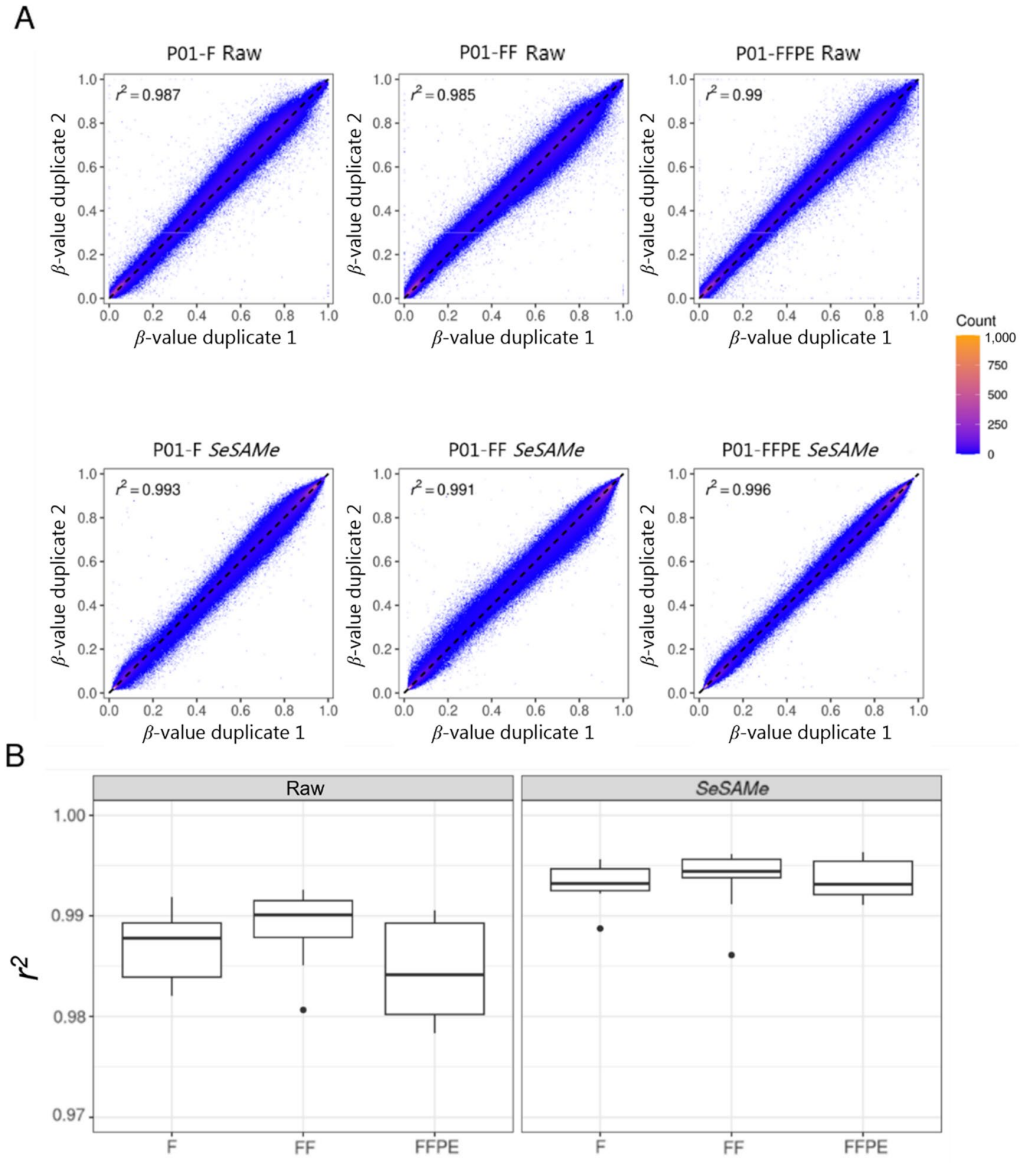
Correlations between duplicate *ß*-values of fresh, frozen, and FFPE tissues. (**A**) Scatter plots of duplicate *ß*-values of fresh, frozen, and FFPE tissue for patient 1*.* (**B**) Box plots of the coefficient of correlations (*r*^*2*^) of all duplicates*.* F = Fresh tissue, FF = Frozen tissue, FFPE = Formalin-fixed, paraffin-embedded tissue.

To assess the reproducibility of EPIC array *ß*-values of fresh, frozen, and FFPE tissues, the coefficients of correlation (*r*^*2*^) between duplicate *ß*-values were calculated for all patients (Fig. 5A, Supplementary Figure S2). The duplicate raw* ß*-values correlated with a median *r*^*2*^ of: fresh = 0.988, frozen = 0.990, and FFPE = 0.984. The correlation of the *SeSAMe ß*-values was higher than that of the raw *ß*-values with a median *r*^*2*^ of: Fresh = 0.993, frozen = 0.994, FFPE = 0.992 (Fig. 5B).

**Figure 6:**
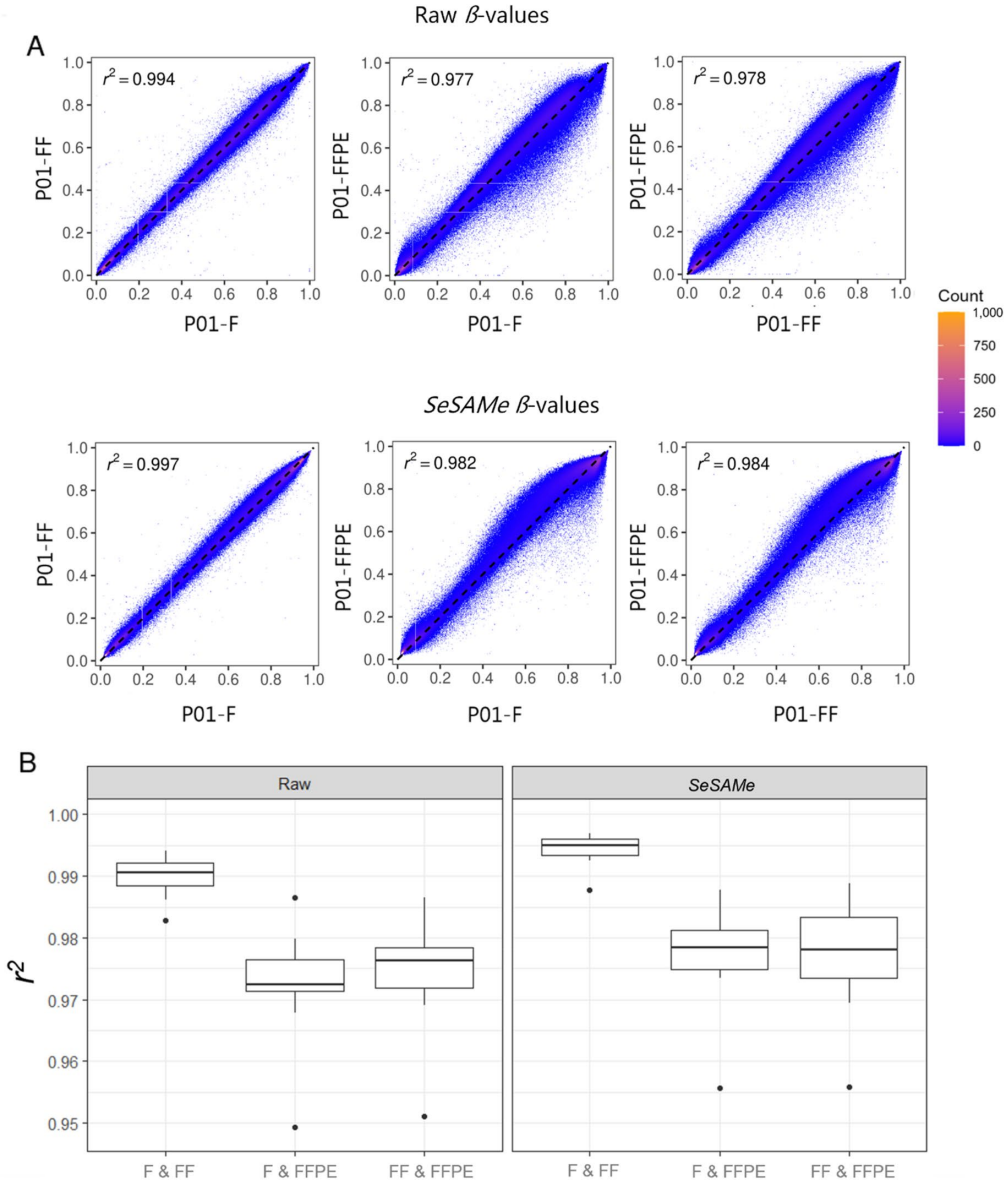
Correlations between paired DNA methylation in fresh, frozen, and FFPE tissues. (A) Scatter plots of paired *ß*-values (means of duplicates) between fresh, frozen, and FFPE samples from patient 1*.* (B) Box plots of the coefficients of correlation (*r*^*2*^) among all storage methods, bottom outliers in “F & FFPE” and “FF & FFPE” are patient 2 for both Raw and *SeSAMe*. F = Fresh tissue, FF = Frozen tissue, FFPE = Formalin-fixed, paraffin-embedded tissue.

To investigate the correlation of DNA methylation between the storage methods, the coefficients of correlation (*r*^*2*^) between *ß*-values of fresh, frozen, and FFPE samples were calculated (Fig. 6A, Supplementary Figure S3). The *ß*-values of fresh and frozen tissues were highly correlated (Raw: median *r*^*2*^ = 0.991; *SeSAMe*: median* r*^*2*^ = 0.995). The correlations between FFPE tissue and fresh and frozen tissues were slightly lower with raw *ß*-values (FFPE vs. fresh, median *r*^*2*^ = 0.972. FFPE vs. frozen, median* r*^*2*^ = 0.976) than with *SeSAMe ß*-values (FFPE vs. fresh, median *r*^*2*^ = 0.978. FFPE vs. frozen, median *r*^*2*^ = 0.977) (Fig. 6B).

**Figure 7:**
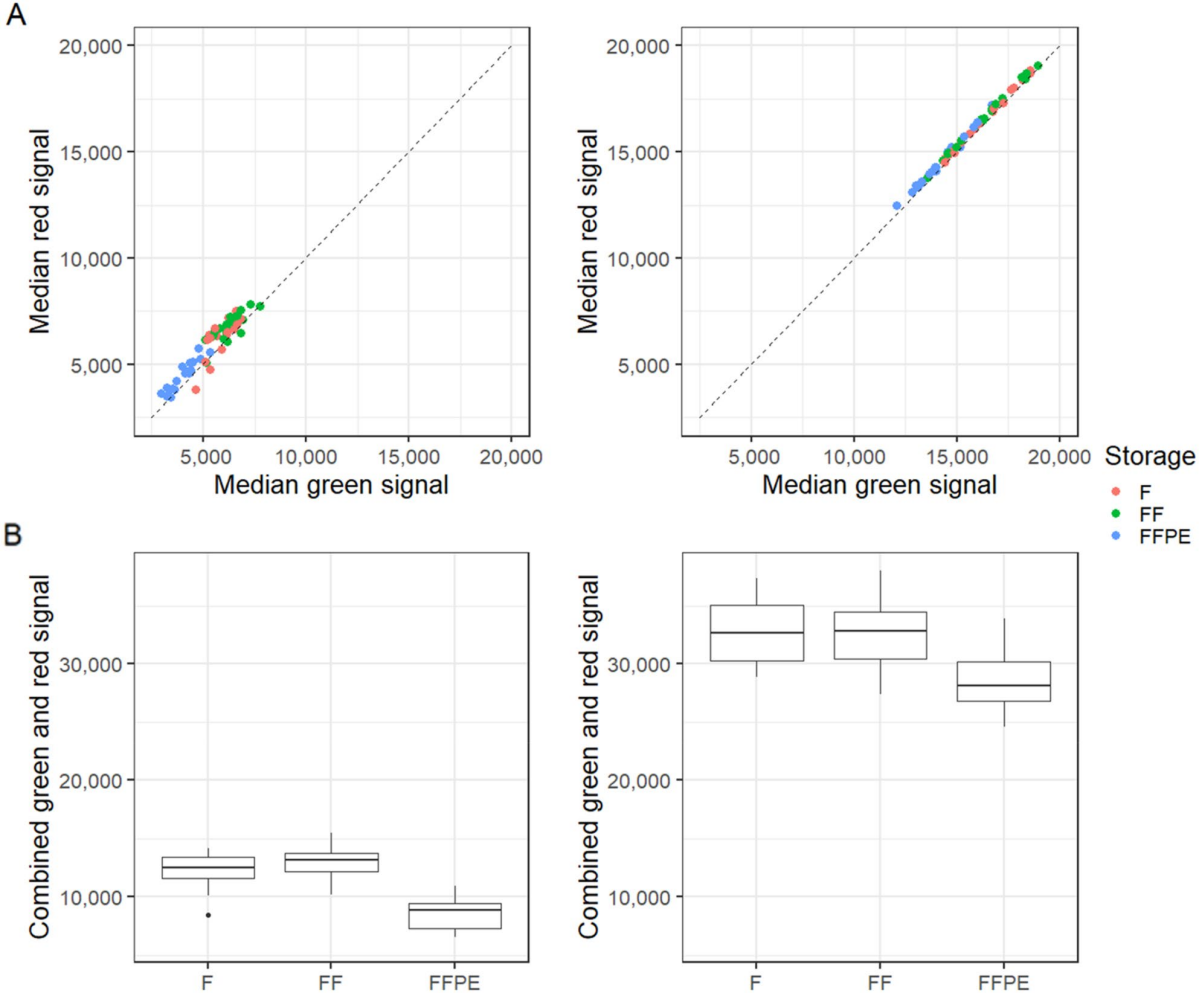
Median red and green signal intensities with the EPIC array. (**A**) Scatter plots of per-sample median green and red signals for Raw and *SeSAMe* data (n = 54, duplicates of 9 patient samples in each group). (**B**) Box plots of combined per-sample median green and red signals for Raw and *SeSAMe* data, be aware of different (n = 54, duplicates of 9 patient samples in each group). F = Fresh tissue, FF = Frozen tissue, and FFPE = Formalin-fixed, paraffin-embedded tissue.

To further investigate how the storage conditions affected the EPIC array data, we investigated the green and red signal intensities. When the Intensity DATa files (.IDAT) were loaded into R as raw data, the per-sample correlations between the median green and red signals of fresh, frozen, and FFPE tissues were: Fresh: *r*^2^ = 0.649, frozen: *r*^2^ = 0.640, and FFPE tissues: *r*^2^ = 0.881 (Fig. 7A). When the .IDAT files were loaded into R using *SeSAMe*, the per-sample correlations between median green and red signals of fresh, frozen, and FPPE tissues were: Fresh: *r*^2^ = 0.996, frozen: *r*^2^ = 0.998, and FFPE: *r*^2^ = 0.993 (Fig. 7A). The combined median green and red signal intensities of the raw data ranged between 6,593 and 15,476 (mean fresh = 12,249, frozen = 13,047, FFPE = 8,520) (Fig. 7B). With *SeSAMe*, the combined median signal intensities ranged between 24,565 and 37,984 (median fresh = 32,892, frozen = 32,750, and FFPE = 28,647) (Fig. 7B).

**Figure 8:**
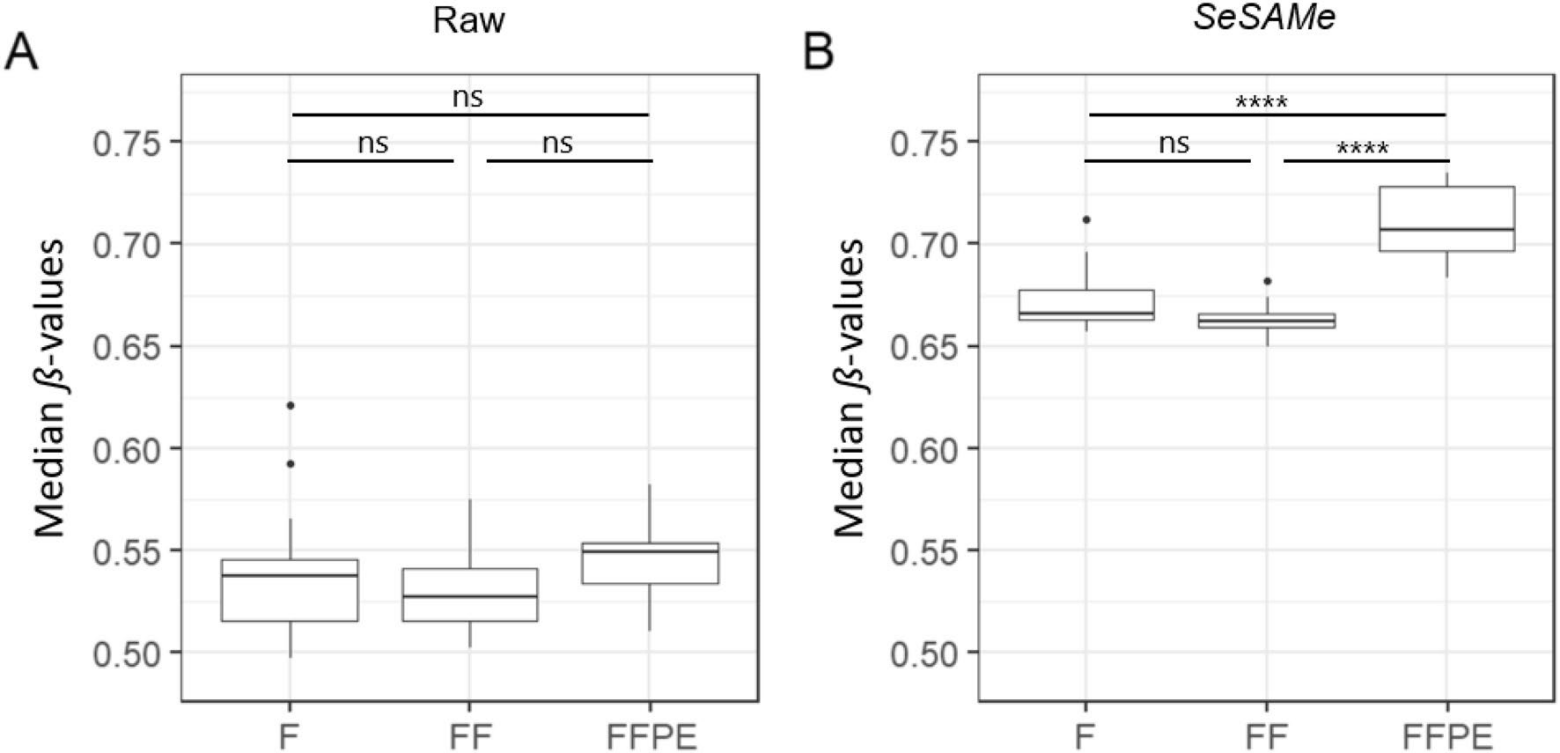
Per-sample median *ß*-values. Box plots for each storage method of the per-sample median *ß*-values for Raw (**A**) (n = 54, duplicates of 9 patient samples in each group) and *SeSAMe* (**B**) (n = 54, duplicates of 9 patient samples in each group). F = Fresh tissue, FF = Frozen tissue, FFPE = Formalin-fixed, paraffin-embedded tissue. Paired students t-tests, ns = non significant, **** = p < 0.0001.

The median *ß*-values of FFPE tissue were higher than those of fresh and frozen tissues (Fig. 8). The result was not statistically significant (p > 0.05) for the raw *ß*-values (means of fresh = 0.54, frozen = 0.53, and FFPE = 0.55) (Fig. 8A). The *SeSAMe ß*-values of the FFPE tissue (mean = 0.71) were statistically significantly higher than those of fresh (mean = 0.67, p < 1 × 10^–4^) and frozen tissues (mean = 0.66, p < 1 × 10^–4^). On average, the median *SeSAMe ß*-values of FFPE tissues were 0.06 (6.0%) and 0.07 (7.0%) higher than those of fresh and frozen tissues. Within patients, an average of 64.1% and 62.9% of the CpG sites of FFPE tissues had higher *ß*-values than those of fresh and frozen tissues, respectively (Supplementary Figure S4). When comparing fresh and frozen tissues an average of 51.3% of the CpG sites had higher *ß*-values in fresh than frozen tissue. *ß*-values of 612,493 CpG sites were obtained from all samples. 131,025 (21.4%) of these CpG sites had higher *ß*-values in FFPE tissue than fresh and frozen tissue in all patients, and 34,806 (5.7%) of the CpG sites had lower *ß*-values in FFPE tissue than fresh and frozen tissue. Of the 131,025 CpG sites with increased *ß*-values in FFPE tissue, 89.6% were hybridized with Type II probes, and 11.8% were located in CpG islands. Among all CpG sites detected, 83.6% were hybridized with Type II probes, and 18.6% were located in CpG islands (Supplementary Table S2 & S3).

## Discussion

Biopsies from surgeries and autopsies are mainly stored by FFPE or freezing. To investigate if the method of storage affected DNA methylation, paired cardiac tissues of fresh, frozen and FFPE were analysed using the genome wide EPIC array methylation chip. We found that the DNA from FFPE had lower quality (mean DI = 2.51, mean ΔCt = 2.03) than DNA extracted from frozen (mean DI = 0.84, mean ΔCt = − 1.46) and fresh (mean DI = 0.97, mean ΔCt = − 1.47) cardiac tissues. We observed no negative effect on the DNA quality by freezing the tissue compared to fresh tissue, supporting that freezing (− 80°C) of tissue is suitable for preserving the DNA.

Multiple analysis pipelines exist to analyse the signal intensity data obtained by the EPIC array. In this study, we examined the raw data (*minfi* pipeline with no pre-processing of data) and normalized the data with the *SeSAMe* pipeline (standard settings), including probe masking due to poor design and detection p-value, dye bias correction, and background subtraction. After in silico QC with *SeSAMe*, we found that samples with low DNA quality (high DI), i.e., the FFPE samples, had more CpG probe data removed than fresh and frozen samples*.* This observation was in line with our previous study, where we investigated the correlation between the DNA sample quality of FFPE samples and the number of EPIC array probes usable for subsequent analysis^21^. A likely explanation is the generally lower CpG probe signals of FFPE tissue (Fig. 7), leading to decreased signal-to-noise ratios resulting in increased detection p-values and increased removal of CpG probe data.

The *ß*-values of the FFPE tissue samples clustered separately from the fresh and frozen tissue samples. This is in line with other studies investigating DNA methylation levels in frozen and FFPE tumour tissues^16–18^. Similar clusterings were also found for gene expression levels in differently stored (fresh, frozen, and FFPE) cardiac tissues^22^. Furthermore, the clustering of samples fitted well with our correlation analyses of the DNA methylation patterns. Here, high correlations (*r*^2^) were found with all storage methods when comparing the DNA methylation between tissue sample duplicates. This indicates good reproducibility for all storage methods, comparable to that observed in other tissues^23^.

The DNA methylation level of FFPE tissue were biased towards higher *ß*-values with the median *SeSAMe ß*-values of FFPE tissue on average being 6.0% and 7.0% higher than those of fresh and frozen tissues, respectively. This indicates that the average DNA methylation level obtained with FFPE tissue is overestimated when assessed with *SeSAMe*. The difference was not caused by a few CpG *ß*-values being highly overestimated, but rather by a global trend with 64.1% and 62.9% of the CpG sites having higher *ß*-values in FFPE than in fresh and frozen tissues. By chance about 50% of the probes would be expected to be higher in one tissue over the other, as seen with fresh and frozen tissue, where 51.3% of the CpG sites were found to have higher *ß*-values in fresh compared to frozen tissue. Furthermore, 21% of the CpG sites showed a systematic overestimation of the *ß*-values in FFPE samples compared to fresh and frozen tissues. The DNA methylation of fresh and frozen tissues from the same individual were highly correlated. Thus, freezing does not appear to affect the DNA methylation pattern of the tissue and can be seen as an accurate representation of the DNA methylation status at the time of collection.

The differences in *ß*-values obtained from FFPE tissues compared to fresh and frozen tissues were most likely caused by the destructive nature of formalin. However, the DNA extraction protocol for the FFPE tissue was slightly different from that of fresh and frozen tissue, including additional steps for removing paraffin from the tissue and restoring DNA. Thus, the extraction method could be a source of variation in the data. The FFPE restoration is a necessary step and choosing an alternative FFPE restoration method does not improve the comparability of the *ß*-values of FFPE tissue and frozen tissue^16^. The storage time of the samples in this study was relatively short compared to those found in pathology archives world wide^24,25^. Some studies have shown that DNA degradation was increased with storage time, whereas other studies showed that the storage time only had a minor or no effect on the DNA quality^25–29^. Although the storage time of a sample can be a rough indicator of DNA quality, the utility of archived FFPE tissue for DNA methylation analysis appears to be more dependent on the assessed DNA quality than the storage time^21,24,29^. This could be due to factors such as the tissue quality before storage and the humidity and temperature of the FFPE storage facilities.

As observed in other studies, the median *ß*-values were higher after *SeSAMe* analysis of the EPIC array data than after raw analysis of the EPIC array data^30^. We found the difference between raw median *ß*-values and the *SeSAMe* median *ß*-value to be larger for FFPE tissue than for fresh or frozen tissues (Fig. 8). This is likely why the observed overestimation of *ß*-values in FFPE is more profound and only statistically significant when the EPIC array data is analysed by *SeSAMe*. A likely cause to the higher *ß*-value difference in FFPE samples is the lower DNA quality of the FFPE tissue. Degraded DNA can be a cause of impaired hybridisation of the target DNA to the probes of the EPIC array and may result in altered signal-to-noise ratio and dye bias. Thus, the FFPE tissue samples were corrected for more noise and bias by the SeSAMe pipeline than the fresh and frozen tissue samples were.

Based on the data presented here, we found that the DNA methylation results from frozen samples (-80°C) were similar to those of fresh tissue. The DNA methylation levels from FFPE tissues were higher than those obtained from fresh and frozen tissues, highlighting that DNA methylation levels may be overestimated in FFPE tissue samples. Therefore, we advise against direct comparisons of DNA methylation results from FFPE tissue and fresh and frozen tissues.

## Materials and methods

### Ethics

The study conformed to the declaration of Helsinki and was approved by the Committees on Health Research Ethics in the Capital Region of Denmark (H-20039524). The biobank where the samples are held is registered at the University of Copenhagen’s joint records of processing of personal data in research projects and biobanks (514-0528/20-3000) and complies with the rules of the General Data Protection Regulation (Regulation (EU) 2016/679). Informed written consent was obtained from all individuals. Patient data were pseudonymized.

### Tissue collection

The tissue used for this study was collected and treated as previously descriped^22^. Tissue from the right atrial appendage (RAA) was collected from 10 individuals undergoing scheduled cardiac surgery at Rigshospitalet, Copenhagen, Denmark. Due to a low amount of tissue, patient five was excluded from further analysis. Descriptive data on the patients included in the study are presented in Supplementary Table S1. Each fresh RAA sample was divided into three pieces for DNA extraction: 1. DNA extraction immediately after tissue collection (fresh tissue), 2. Tissue was frozen at -80 °C (frozen tissue), or 3. Formalin-fixation and paraffin-embedding (FFPE tissue). The median time from tissue collection to extraction/freezing/fixation was 27 min. (range: 14–41 min.). A graphical presentation of the workflow is presented in Fig. 1.

### Formalin-fixed and paraffin-embedded samples

RAA tissue samples were fixed with 4% buffered formaldehyde (10% buffered formalin) using the BiopSafe Biopsy Sample System (BiopSafe, Denmark). Fixation times ranged from 23 to 97 h (median: 69 h). Tissues were dehydrated and paraffin treated using a Tissue-Tek VIP 6 AI (Sakura Finetek Europe, the Netherlands) and included the following incubations: 1 × 4% buffered formaldehyde for 60 min., 6 × EtOH for 90 min. with increasing concentrations of EtOH, 2 × Histolab Clear (Histolab Products AB, Sweden) for 60 min., 1 × Histolab Clear for 120 min., and 4 × Paraffin for 80 min. Lastly, the tissues were embedded in paraffin.

### DNA extraction

DNA from fresh and frozen tissues was extracted using the Dneasy Blood & Tissue Kit (Qiagen, Germany) following the manufacturer’s recommendations. Approximately 5 mm^3^ of tissue was homogenised for 2 × 2 min at 20 Hz using the TissueLyser II (Qiagen, Germany). The DNA was eluted in 100 µL provided buffer AE. Frozen tissue samples were stored at -80°C for a median time of 94 days (range: 91–107 days) before DNA extraction. DNA from FFPE tissue was extracted using the QIAamp® DNA FFPE Tissue kit (Qiagen, Germany). The manufacturer’s recommendations were followed with the exception that Proteinase K digestion was conducted overnight until the tissue was completely dissolved. A total of 5 × 20 µm slides with approximately 7 × 7 mm tissue was used per extraction. The paraffin was removed with 1ml xylene followed by a wash in 96–100% ethanol. The DNA was eluted in 55 µL of the provided buffer ATE. The FFPE tissue was stored at room temperature for a median time of 105 days (range: 98–119 days) before DNA extraction. DNA extractions from the nine patients were performed in duplicates. The quantity of the DNA was measured using the Qubit dsDNA HR assay kit (Thermo Fisher Scientific, Waltham, MA, USA).

### DNA quality assessment

*Infinium QC*. The quality of DNA from each sample was assessed with the Infinium HD FFPE QC kit (Illumina, Inc., CA, USA) using an ABI 7900 (Thermo Fisher Scientific, Waltham, MA, USA) following the manufacturer’s protocol. The qPCR cycle thresholds (Ct) were used to calculate the ΔCt = Ct(sample) – Ct(QCT), where Ct(sample) is the Ct of the tissue sample, and Ct(QCT) is the Ct value of the Quality Control Template DNA (QCT) provided by Illumina. The primer set generates an amplicon between 175 and 200 bp in length. Illumina considers DNA from FFPE tissue with a ΔCt < 5 eligible for further analysis with the EPIC array.

*Quantifiler Trio.* The quality of DNA from each sample was assessed with the Quantifiler Trio® DNA Quantification kit (Thermo Fisher Scientific, Waltham, MA, USA) using an ABI 7900 (Thermo Fisher Scientific, Waltham, MA, USA) following the manufacturer’s protocol. Quantifiler Trio uses three primer sets to generate a small (80 bp), a large (214 bp) autosomal amplicon, and a Y-chromosomal amplicon (74 bp) together with an internal PCR control amplicon (IPC). The ratio between the smaller and larger autosomal amplicons, the degradation index (DI), was used to measure the DNA quality. A DI ≤ 1 indicates no degradation, a DI from 1 to 10 indicates moderate degradation or inhibition of the PCR, and a DI > 10 indicates severe degradation of the DNA or inhibition of the PCR (Thermo Fisher Scientific, Waltham, MA, USA, 2018).

### The DNA methylation array

Bisulfite conversion was performed using the EZ DNA Methylation kit (Zymo Research Corp, CA, USA) following the manufacturer’s protocol with 500 ng DNA as input. The converted DNA was eluted in 10 µL elution buffer. DNA extracted from FFPE tissue was restored using the Infinium HD FFPE DNA Restore kit (Illumina, Inc., CA, USA) following the manufacturer’s protocol. The DNA methylation levels were quantified using the Infinium MethylationEPIC kit (Illumina, Inc., CA, USA) following the manufacturer’s protocol. The prepared slides were scanned using the iScan System (Illumina, Inc., CA, USA).

### Data analysis

The data analysis was conducted in the R statistical environment (R version 4.1.1.) using the *tidyverse* package^32^. The raw iScan data (IDAT files) were imported into R using the Bioconductor^33^ packages *minfi*^20^ version 1.44 and *SeSAMe*^19^ version 1.16.1. The plots were made with *ggplot2* in R.

The raw data was assessed with *minfi* without normalization of the red and green colour intensities (RGChannel object) using the *preprocessRaw()* function before the *β*-values were calculated with the *getBeta()* function. QC included the removal of data from probes with the *detectionP()* function of *minfi* (detection p-value > 0.01), probes cross-hybridising with common SNPs (function *dropLociwithSNPs()*), and probes known to hybridize to other segments in the genome^8^.

With *SeSAMe, β*-values and quality metrics, such as median red and green signals and numbers of masked probes, were generated using the function *openSesame()*. The selected pre-processing functions “qualityMask”, “infiniumIChannel”, “dyeBiasNL”, “pOOBAH”, and “noob” remove the data of probes of poor-quality design and signal (detection p-value), correct for dye bias and channel switching, and implement a background subtraction^19^*.*

The *IlluminaHumanMethylationEPICanno.ilm10b5.*hg38^33^ package was used to characterize the probes as Type I or Type II and locate the CpG positions relative to the CpG Islands.

### Statistics

Pearson’s correlation coefficient (r^2^) was calculated for the *β*-values of the sample duplicates and the sample duplicate means of tissue comparisons.

Student’s paired and unpaired t-tests were performed with *t.test()* to identify statistically significant differences in DNA quality and median β-values among fresh, frozen, and FFPE tissues.

Principal component analysis (PCA) was conducted on duplicate mean β-values of all CpG sites with data in all samples using the *prcomp()* function in the *limma*-package version 3.52.4.

Wilcoxon’s rank-sum tests was performed with *wilcox.test()* to identify statistically significant differences in the number of CpG sites, whose data were removed by QC, among fresh, frozen, and FFPE tissue.

### Supplementary Information

Supplementary Information.

## Data Availability

The datasets used and/or analysed during the current study are available from the corresponding author on reasonable request.
