# Supplementary material for: Fresh and frozen cardiac tissue are comparable in DNA methylation array β-values, but formalin-fixed, paraffin-embedded tissue may overestimate DNA methylation levels
Source: Sci Rep. 2023 Sep 29;13:16381. doi: 10.1038/s41598-023-43788-2 (PMC10541404; doi:10.1038/s41598-023-43788-2)
Supplement: Supplementary file 1 — Supplementary Information. [file 41598_2023_43788_MOESM1_ESM.pdf]

Fresh and frozen cardiac tissue are comparable in DNA methylation array β-values, but formalin-fixed, paraffin-embedded tissue may overestimate DNA methylation levels

Mikkel Eriksen Dupont[1*], Stine Bøttcher Jacobsen[1], Steffan Noe Niikanoff Christiansen[1,2], Jacob Tfelt-Hansen[1,3], Morten Holdgaard Smerup[4], Jeppe Dyrberg Andersen[1‡] & Niels Morling[1‡].

1 Section of Forensic Genetics, Department of Forensic Medicine, Faculty of Health and Medical Sciences, University of Copenhagen, Copenhagen, Denmark, **2** Department of Health Science and Technology, Aalborg University, Aalborg, Denmark, **3** Department of Cardiology, Rigshospitalet, Copenhagen University Hospital, Copenhagen, Denmark, **4** Department of Cardiothoracic Surgery, Rigshospitalet, Copenhagen University Hospital, Copenhagen, Denmark. Email: forensic.genetics@sund.ku.dk

‡ These authors contributed equally to this work and are joint senior authors.

* Corresponding author: mdupont@sund.ku.dk

Supplementary material

Supplementary Table S1. Descriptive characteristics of the study population.

**Characteristics**	**n = 9**
Male sex, n (%)	9 (100%)
Median age at the time of tissue collection, years (range)	67 (50-80)
Type of surgery performed*, n (%)	
CABG	7 (77.8%)
Valve repair/replacement	4 (44.4%)
History of atrial fibrillation, n (%)	
Before surgery	1 (11.1%)
During surgery	0 (0.0 %)
After surgery	5 (55.5 %)

* Two patients underwent both coronary artery bypass graft (CABG) and valve repair/replacement surgery.

Supplementary Table S2. Fraction of probes grouped by their locations relative to the CpG Islands.

Relation to CpG island	North Shelf	North Shore	CpG Island	South Shore	South Shelf	Open sea
Fractions of all probes	0.0401	0.0736	0.1865	0.0616	0.0386	0.5638
Fractions of probes with increased *ß*-values in FFPE tissue	0.0369	0.0963	0.1184	0.0822	0.0343	0.6670

Supplementary Table S3. Fractions of Type I and II probes.

Probe type	Type I	Type II
Fraction of all probes	0.1642	0.8358
Fraction of probes with increased *ß*-values in FFPE tissue	0.1042	0.8959

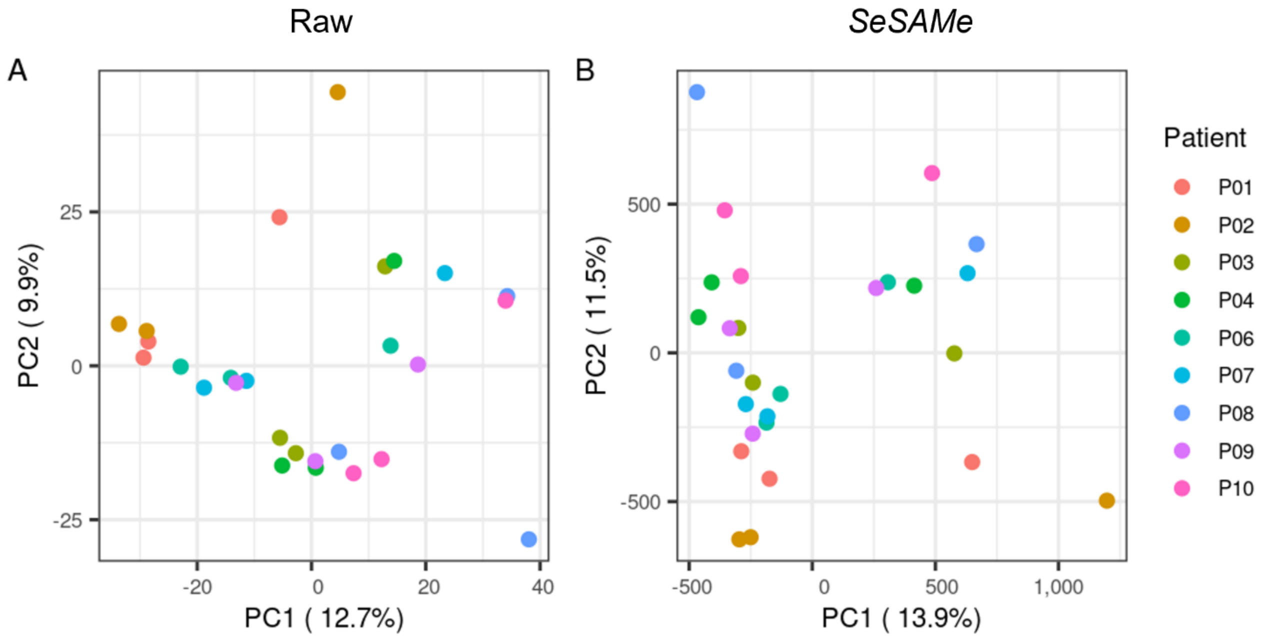

Supplementary Figure S1. Principal component analyses of *ß*-values. Duplicate mean *ß*-values of raw data (A) (n = 27) and *SeSAMe* data (B) (n= 27). Abbreviations: F = Fresh tissue, FF = Frozen tissue, FFPE = Formalin-fixed, paraffin-embedded tissue.

Patient 2

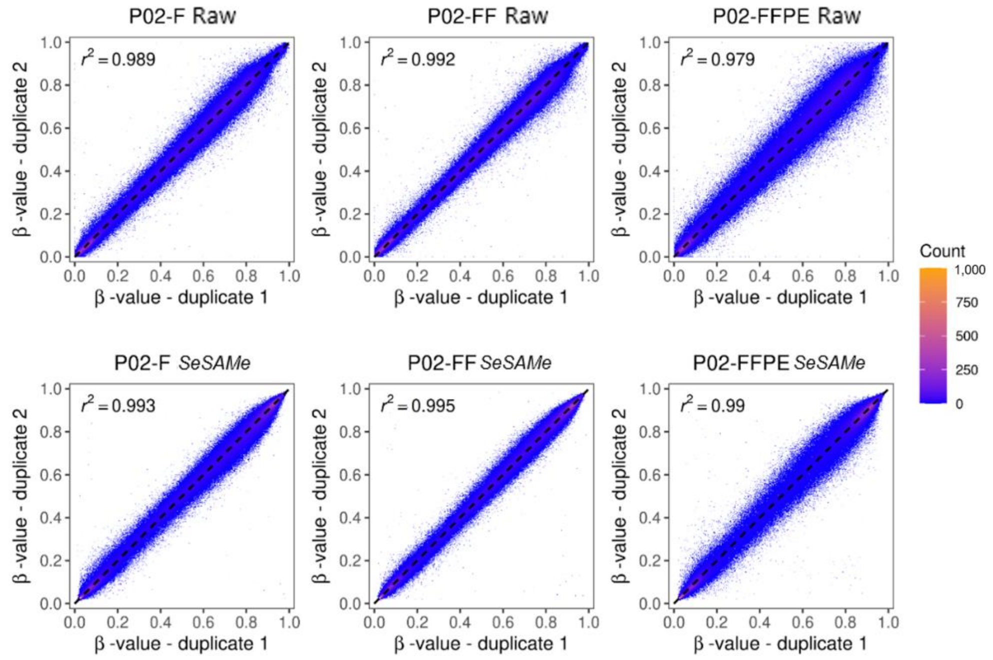

Patient 3

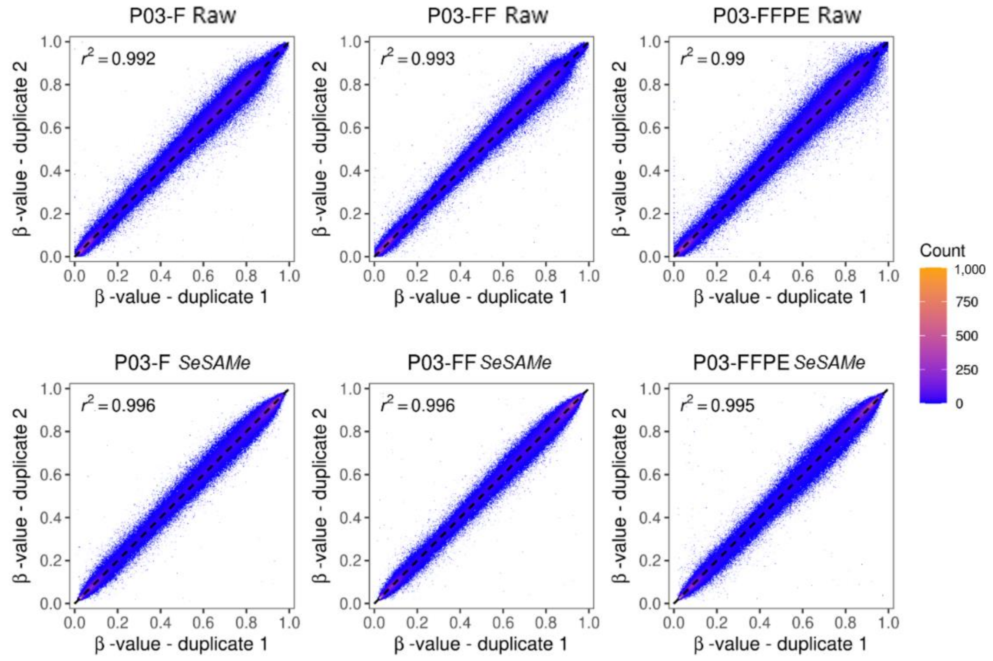

Supplementary Figure S2. Correlations between duplicate *ß*-values of fresh, frozen, and FFPE tissues. Scatter plots of duplicate *ß*-values of fresh, frozen, and FFPE tissue for patients 2-4 and 6-10*.*

Patient 4

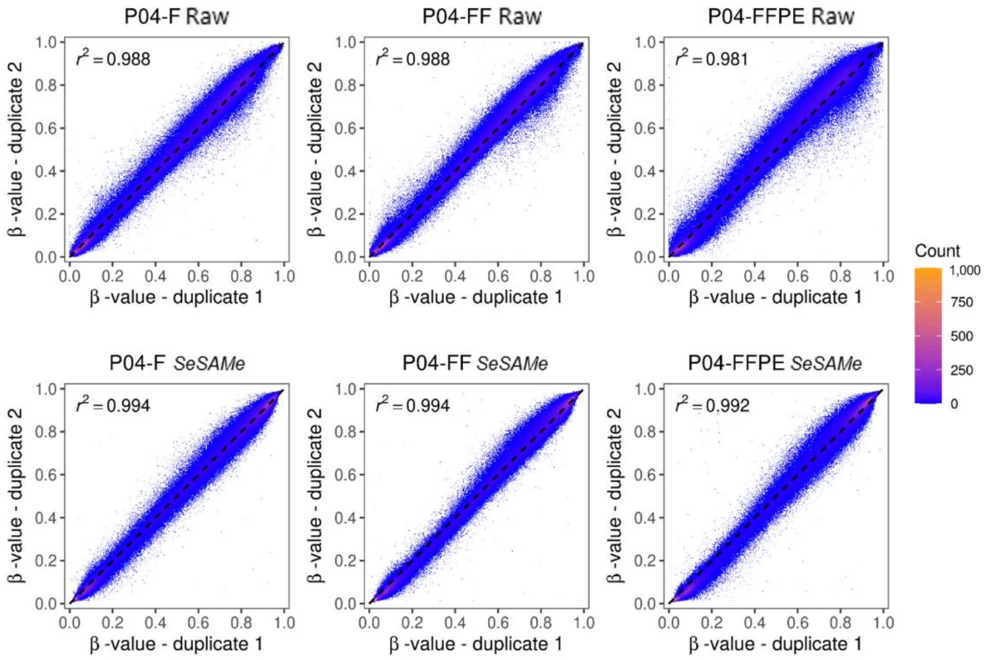

Patient 6

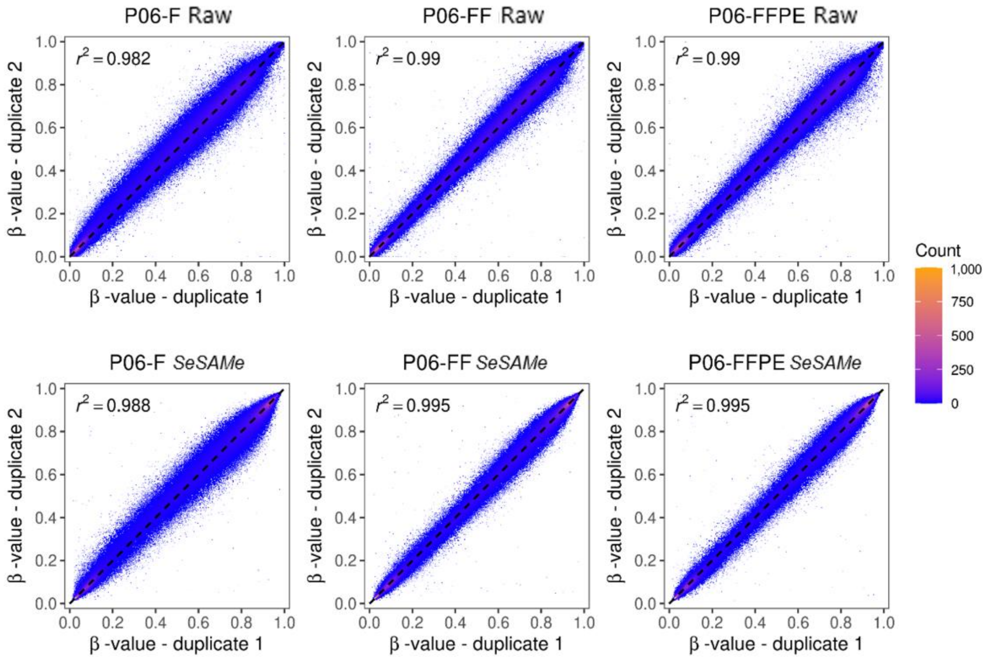

Supplementary Figure S2. Correlations between duplicate *ß*-values of fresh, frozen, and FFPE tissues (continued).

Patient 7

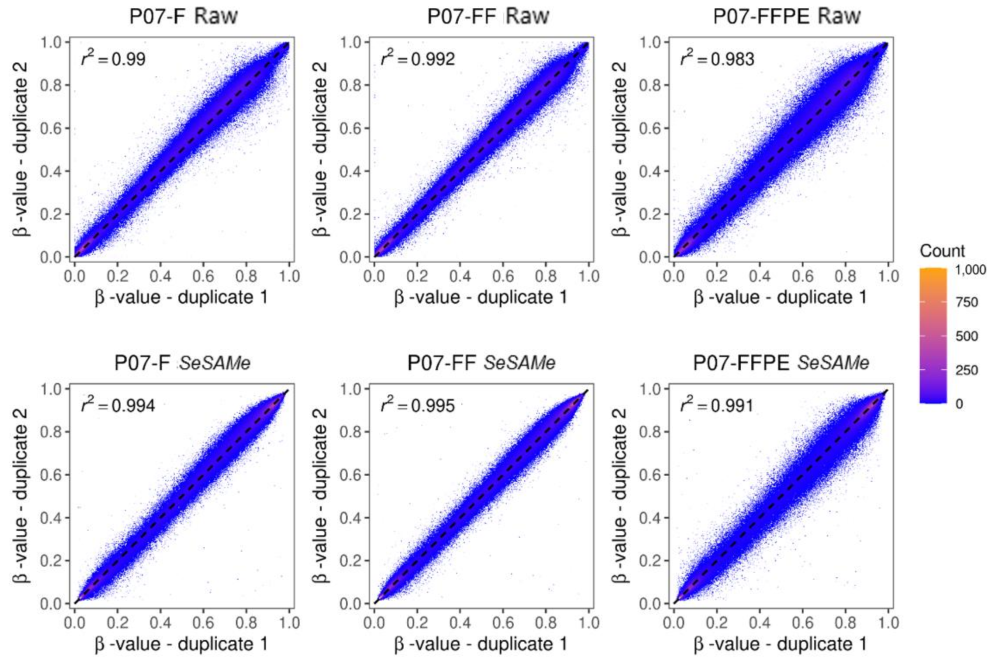

Patient 8

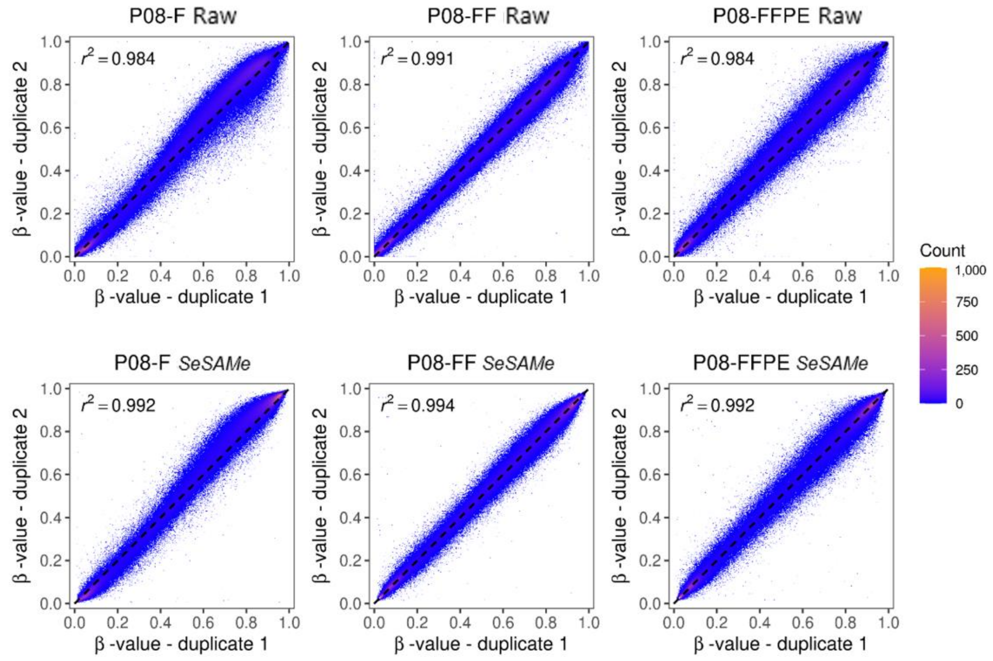

Supplementary Figure S2. Correlations between duplicate *ß*-values of fresh, frozen, and FFPE tissues (continued).

Patient 9

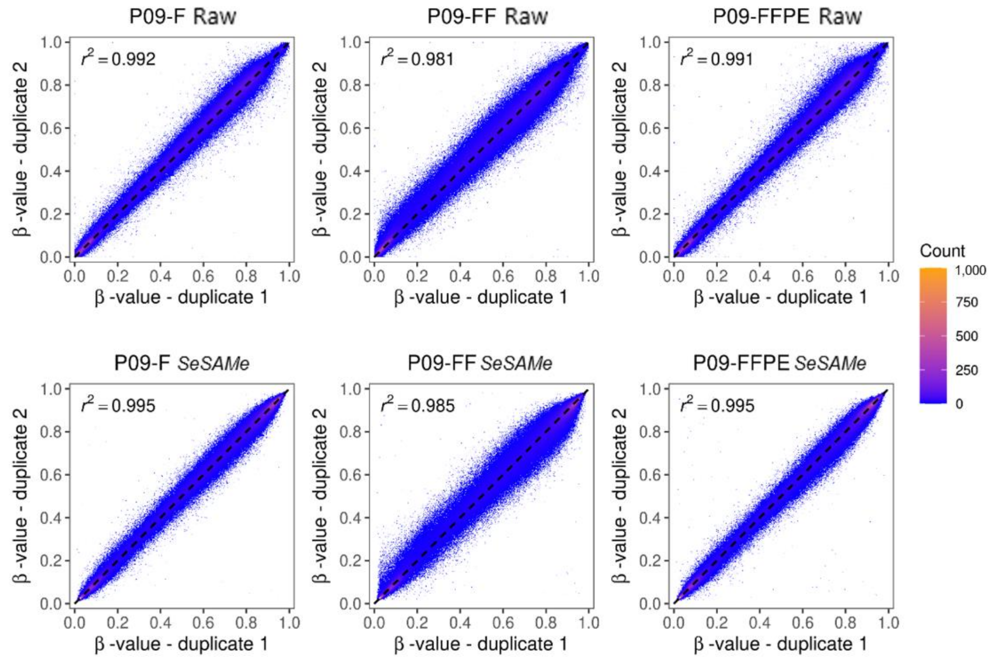

Patient 10

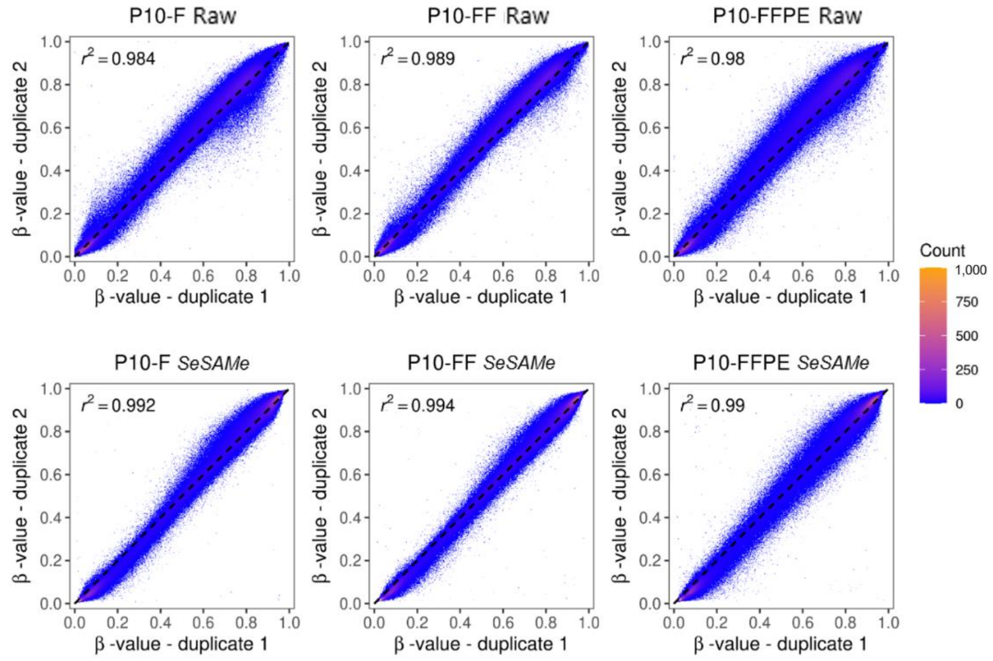

Supplementary Figure S2. Correlations between duplicate *β*-values of fresh, frozen, and FFPE tissues (continued).

Patient 2

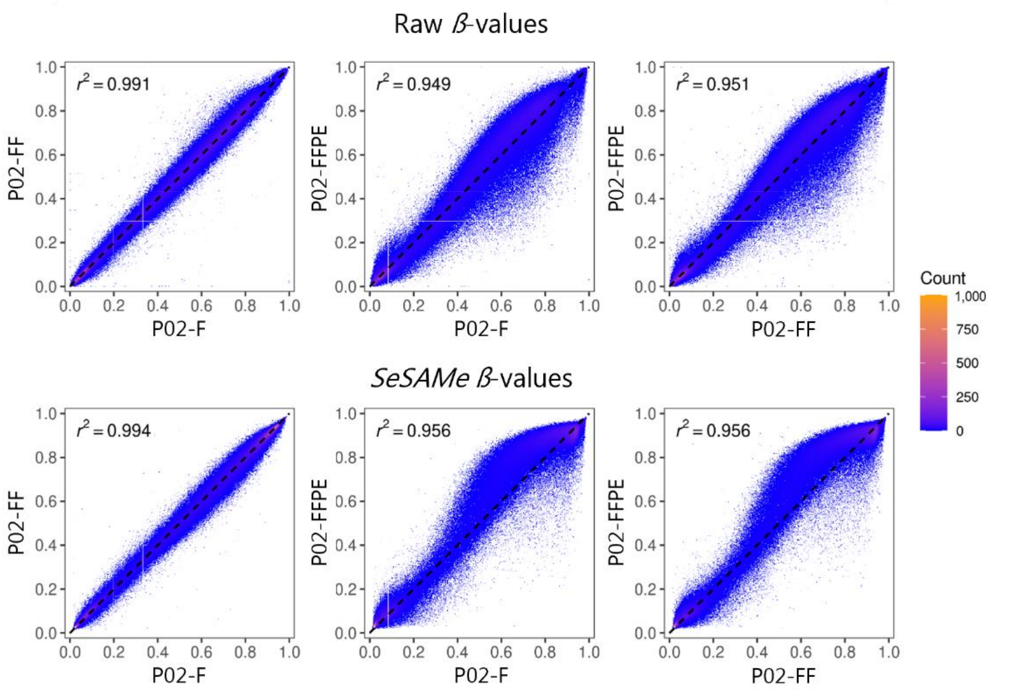

Patient 3

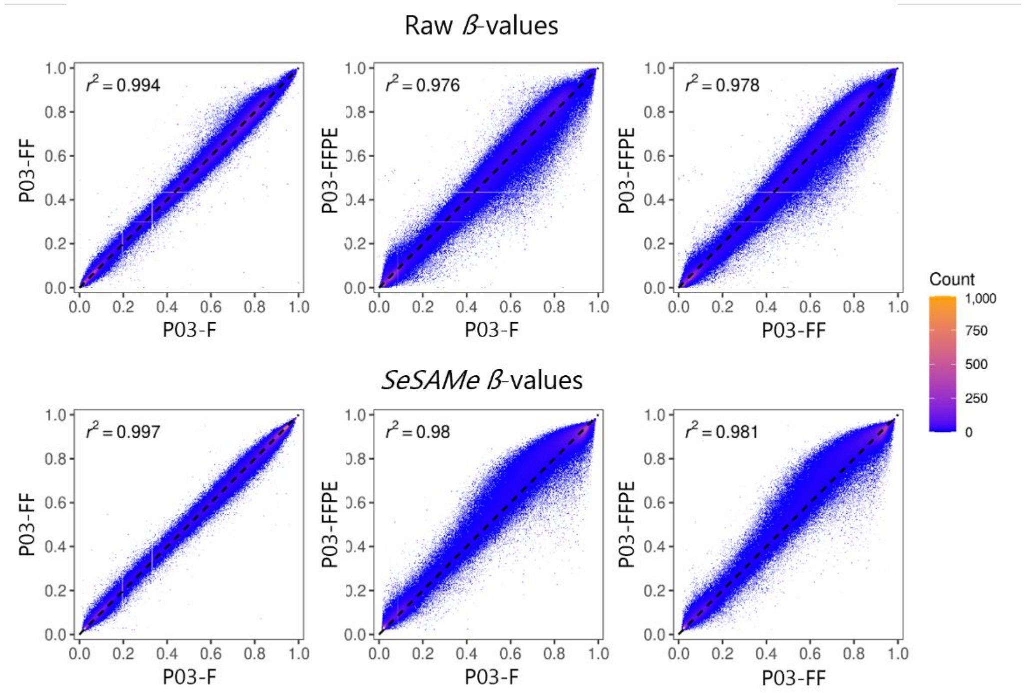

Supplementary Figure S3. Correlations between paired *ß*-values of fresh, frozen, and FFPE tissues. Scatter plots of paired *ß*-values (mean of duplicate) between fresh, frozen, and FFPE samples of patients 2-4 and 6-10.

Patient 4

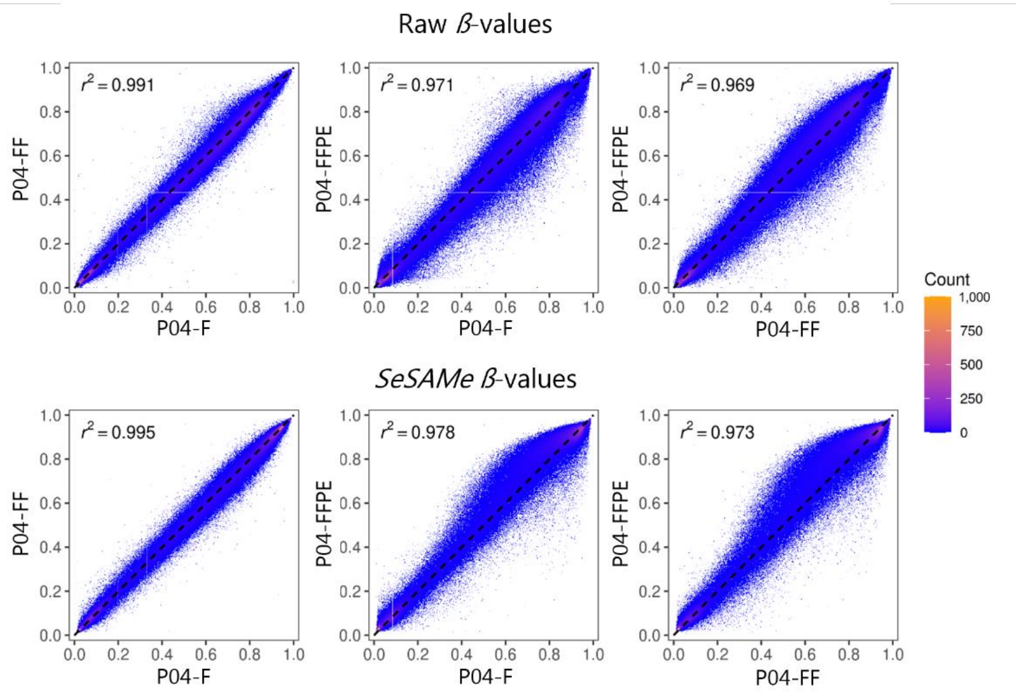

Patient 6

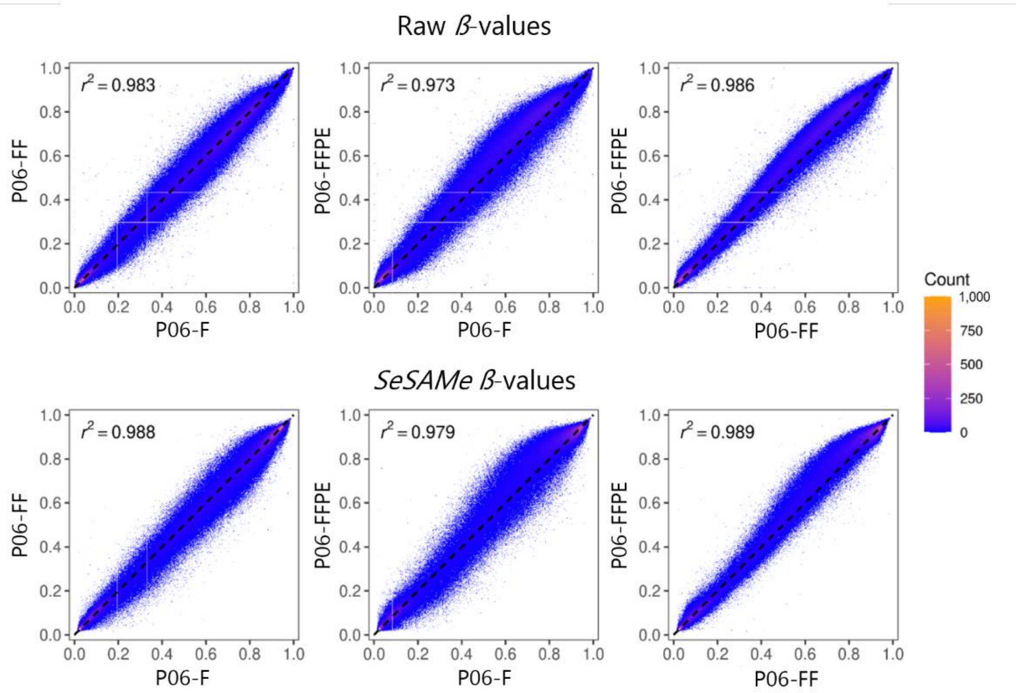

Supplementary Figure S3. Correlations between paired *β*-values of fresh, frozen, and FFPE tissues (continued).

Patient 7

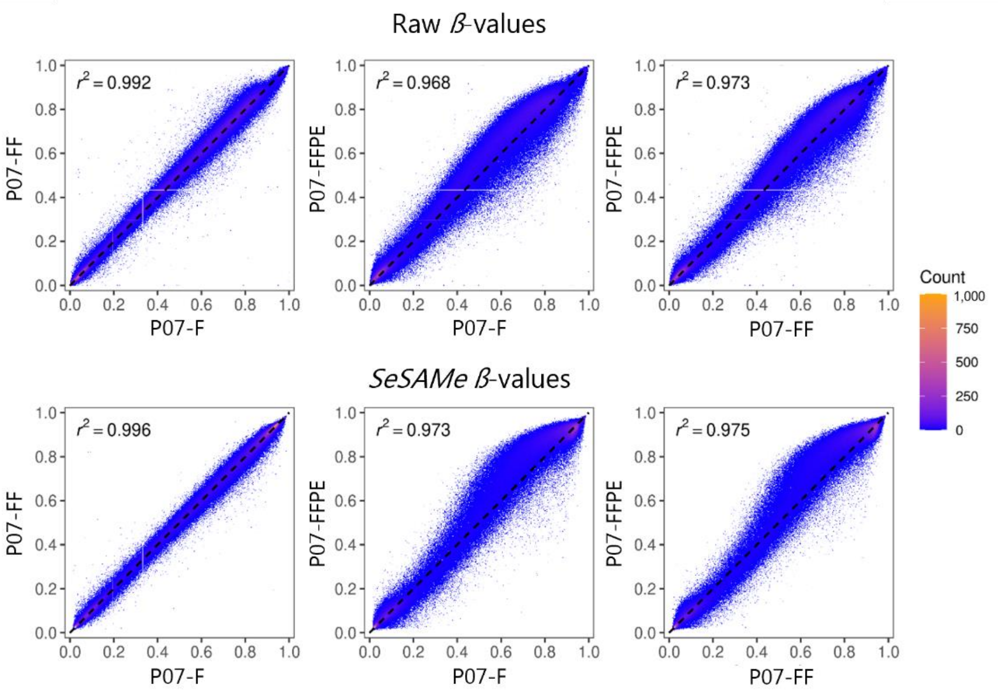

Patient 8

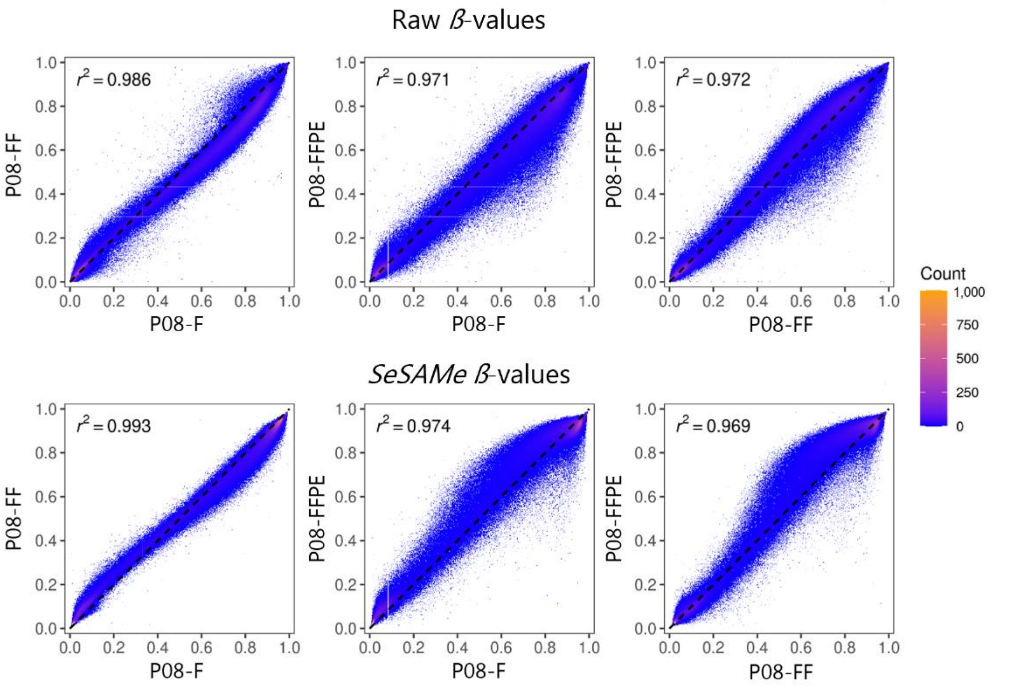

Supplementary Figure S3. Correlations between paired *β*-values of fresh, frozen, and FFPE tissues (continued).

Patient 9

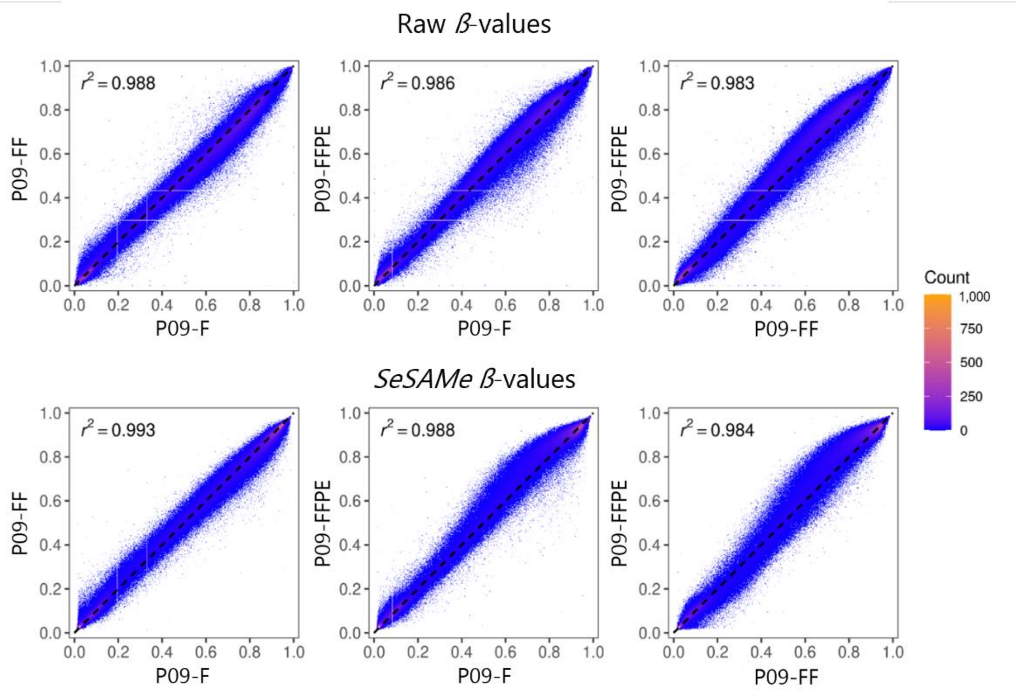

Patient 10

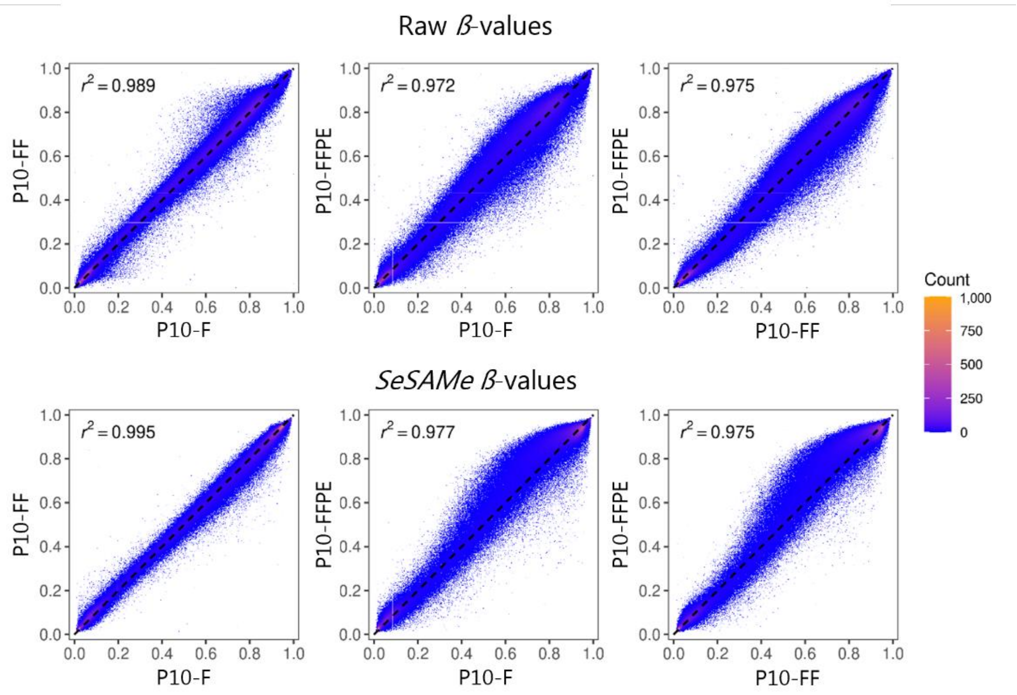

Supplementary Figure S3. Correlations between paired *ß*-values of fresh, frozen, and FFPE tissues (continued).

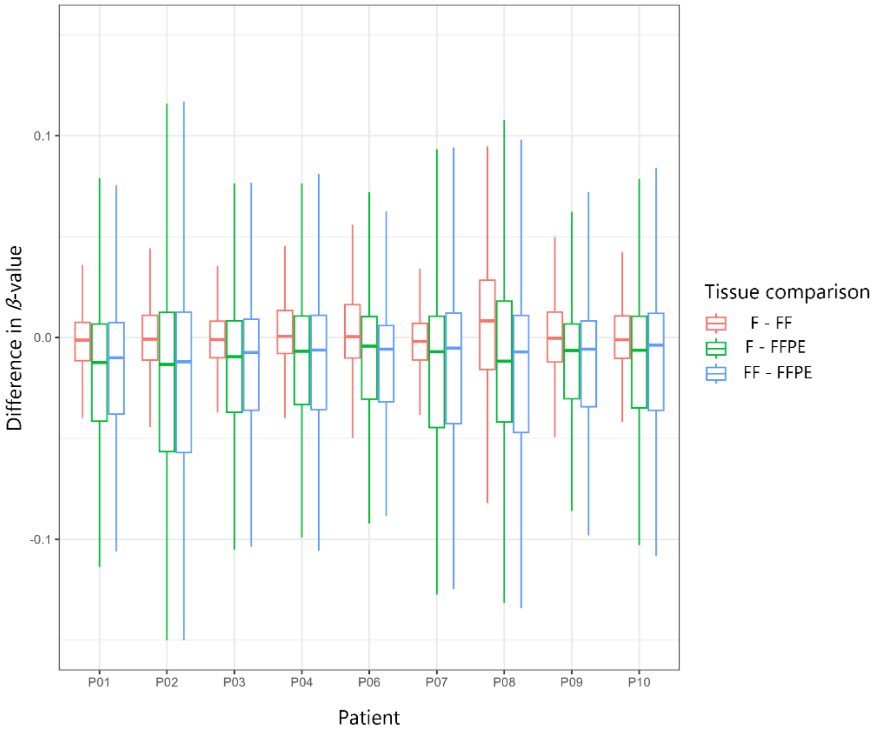

Supplementary Figure S4. Per probe *ß*-value differences. Box plots of the per probe *SeSAMe ß*-value differences among the compared tissue for each patient (outliers were removed from visualization). Abbreviations: F = Fresh tissue, FF = Frozen tissue, FFPE = Formalin-fixed, paraffin-embedded tissue.
